# Therapeutic Potential of Ursonic Acid: Comparison with Ursolic Acid

**DOI:** 10.3390/biom10111505

**Published:** 2020-11-02

**Authors:** Juhyeon Son, Sang Yeol Lee

**Affiliations:** Department of Life Sciences, College of BioNano Technology, Gachon University, Seongnam, Gyeonggi 13120, Korea; wwkk5555@gc.gachon.ac.kr

**Keywords:** ursonic acid, ursolic acid, triterpenoid, anticancer, antiprotozoa, antiproliferation

## Abstract

Plants have been used as drugs to treat human disease for centuries. Ursonic acid (UNA) is a naturally occurring pentacyclic triterpenoid extracted from certain medicinal herbs such as *Ziziphus jujuba*. Since the pharmacological effects and associated mechanisms of UNA are not well-known, in this work, we attempt to introduce the therapeutic potential of UNA with a comparison to ursolic acid (ULA), a well-known secondary metabolite, for beneficial effects. UNA has a keto group at the C-3 position, which may provide a critical difference for the varied biological activities between UNA and ULA. Several studies previously showed that UNA exerts pharmaceutical effects similar to, or stronger than, ULA, with UNA significantly decreasing the survival and proliferation of various types of cancer cells. UNA has potential to exert inhibitory effects in parasitic protozoa that cause several tropical diseases. UNA also exerts other potential effects, including antihyperglycemic, anti-inflammatory, antiviral, and antioxidant activities. Of note, a recent study highlighted the suppressive potential of UNA against severe acute respiratory syndrome coronavirus 2 (SARS-CoV-2). Molecular modifications of UNA may enhance bioavailability, which is crucial for in vivo and clinical studies. In conclusion, UNA has promising potential to be developed in anticancer and antiprotozoan pharmaceuticals. In-depth investigations may increase the possibility of UNA being developed as a novel reagent for chemotherapy.

## 1. Introduction

Historically, plants have long been used to create or obtain medicines to treat a variety of diseases [1]. Medicinal plants are also widely used for chemotherapy treatment of cancer, one of the deadliest groups of disease [2]. Plants can generate a wide range of secondary metabolites, such as alkaloids, terpenes, flavonoids, saponins, and polysaccharides [3]. Although the main roles of plant secondary metabolites are defense from enemies (herbivores, microbes, and other competing plants) and transduction of signals to enhance reproduction, these natural products are also beneficial for humans in terms of treatment of numerous human diseases through regulation of cell metabolism. These mechanisms of therapeutic activities of secondary metabolites include modulation of ion channels, neuroreceptors, enzymatic activity, and cytoskeleton assembly [3,4]. Triterpenoids, a variety of terpene containing 30 carbon atoms, constitute the most diverse group of organic products which can be synthesized by plants [5]. Triterpenoids previously demonstrated anticancer and anti-inflammatory effects in both in vitro and in vivo studies via regulation of transcription factors such as nuclear factor-κB (NF-κB) and STAT3 [5,6].

Among triterpenoids, ursolic acid (ULA) is a widespread secondary metabolite found in pomace, cork, flower, sprout, leaf, and the bark of various plants [7]. ULA is a natural compound well-known for its wide range of beneficial effects, such as anticancer, anti-inflammatory, antibacterial, antiprotozoan, antioxidant, antidiabetic, and antiviral effects [8,9,10,11,12,13]. Ursonic acid (UNA) is a phytochemical which can be also extracted from a great variety of traditional medicinal herbs. Considering that UNA is a triterpenoid which has a molecular structure similar to ULA, it is expected that UNA may demonstrate strong potential to treat human disease as well.

While the therapeutic effects and underlying mechanisms of ULA are well understood, as documented in various scientific studies and reviews, those of UNA are relatively less studied and elucidated. Although ULA and UNA seem quite similar in terms of chemical structure, they are definitely distinct compounds and can exert different biological activities. In this review, we focus on the sanative effects and related cellular mechanisms of UNA. With a comparison between UNA and ULA, we attempt to emphasize the pharmaceutical potential of UNA as a novel drug candidate.

To search the articles regarding UNA and its therapeutic potential, we used PubMed and Google Scholar as databases. The name of UNA and its synonyms were used for keywords to search the literature. We used terms “Ursonic acid”, “3-oxo-urs-12-en-28-oic acid”, “3-oxours-12-en-28-oic acid”, “3-oxo-ursolic acid”, “3-keto ursolic acid”, “UA derivatives” plus “ketone”, “UA derivatives” plus “3-oxo”, “UA acid derivatives” plus “3-keto”, “Ursolic acid derivatives” plus “Ketone”, “Ursolic acid derivatives” plus “3-oxo”, and “Ursolic acid derivatives” plus “3-keto” to search papers relevant to our topic. We included papers from between 1980 and 2020, and there was no restriction on language or type of publication. In total, 22 articles were retrieved from PubMed and 980 publications were found using Google Scholar. We selected papers which conducted experiments regarding extraction or synthesis or the pharmacological potential of UNA. Duplicates and unrelated articles were excluded, and 41 papers were selected for references in regard to UNA.

## 2. Chemical Properties

**Figure 1:**
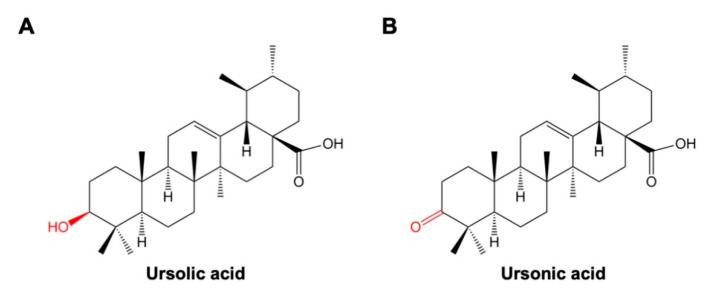
Chemical structures of ursolic acid (ULA) (**A**) and ursonic acid (UNA) (**B**). The only difference between the two molecules is the functional group attached to the C-3 position of ULA and UNA. A β-hydroxy group is attached to the C-3 of ULA, while a keto group is attached to the C-3 position of UNA. This difference may lead to dissimilar bioactivities between ULA and UNA, as shown in several studies.

ULA (3β-hydroxy-urs-12-en-28-oic-acid) is a pentacyclic triterpenoid which has C30H48O3 as its molecular formula and a molecular mass of 456.7 g/mol (Figure 1A) [14]. ULA has low polarity and poor solubility in water. Instead, ULA is highly soluble in organic solvents, including 2-propanol, ethyl acetate, methanol, and ethanol [15]. UNA (3-Oxo-urs-12-en-28-oic acid) is also a polycyclic triterpenoid, with C30H46O3 as its chemical formula and a molecular weight of 454.7 g/mol (Figure 1B) [16]. As polarity of UNA is also low, ethanol and methanol, which are less polar than water, can be used to dissolve UNA [17]. ULA and UNA commonly belong to ursane-type triterpenoids and their basic structures are characterized by five rings, a double bond between C-12 and C-13, and a carboxyl group at the C-28 position, as confirmed by previous NMR studies [18]. Both ULA and UNA hold three oxygen atoms, which can activate ligands and donate lone electron pairs to transition metals [19]. In fact, the carboxyl group at the C-28 position significantly enhances the pharmacological potency of triterpenoids [20,21,22]. The structure of UNA is quite similar to that of ULA, with UNA possessing a keto group at the C-3 position instead of the β-hydroxy group found in ULA. This difference at the C-3 position may be an important factor explaining the dissimilar biological activities of the two compounds.

## 3. Synthesis of Ursonic Acid

Triterpenoids can be synthesized by plants through complicated biochemical pathways [9,23]. In plastids of plants cells, pyruvate (C3) reacts with D-glyceraldehyde 3-phosphate to become the isomers isopentenyl diphosphate (IPP) (C5) and dimethylallyl diphosphate (DMAPP). Synthesis of IPP and DMAPP also occurs in cytosol via decarboxylation of mevalonic acid (C6), which originates from the serial condensation of three units of acetyl-CoA [9]. Condensation reactions of IPP and DMAPP result in the formation of farnesyl diphosphate (C15) and squalene (C30), a precursor of triterpenoids. Several plant enzymes mediate the synthesis of triterpenoids through oxidization, folding, and cyclization of squalene [9,23]. Additional modifications lead to the generation of diverse types of triterpenoids, including ULA and UNA. Since the only difference between UNA and ULA is a functioning group at C-3, UNA can be semisynthesized by oxidation of ULA. Synthesis of UNA can be accomplished by the metabolism of living organisms other than plants. For example, Gram-positive bacteria *Nocardia* species and *Bacillus megaterium* CGMCC 1.1741 was found to biotransform ULA to generate UNA [16,22]. Also, biotransformation of ULA with the fungus *Aspergillus flavus* resulted in isolation of UNA [24]. Moreover, UNA can be synthesized via Jones oxidation, a technique that uses sulfuric acid, chromic trioxide, and acetone to oxidise alcohol [20,25,26,27].

## 4. Plant Sources of Ursonic Acid

UNA can be found in several parts of various plants used in traditional herbal medicines. According to previous studies, UNA can be extracted from fruits of *Crataegus Pinnatifida* and *Malus baccata* [28,29]. In particular, UNA is one of the major compounds present in the ripened fruits of *Ziziphus jujuba*, a main ingredient of traditional Chinese medicinal formula PHY906, which is used for adjuvant therapy of patients with cancer [17,30,31]. It was reported that resins from *Pistacia atlantica, Bursera delpechiana,* and trees of the Dipterocarpaceae family contain triterpenoids, including UNA [32,33,34]. UNA is also found in the roots of *Toona sinensis*, *Piper betle,* and *Ficus microcarpa* [20,35,36]. Extraction of aerial parts of *Lantana camara*, which was traditionally used for treatment of eczema, ulcers, rheumatism, and malaria, also resulted in isolation of UNA [37]. It is known that UNA can be obtained from the leaves of *Lantana tiliaefolia* and *Rauvolfia vomitoria* [38,39]. Furthermore, UNA was isolated from a whole plant of *Catharanthus Roseus* and *Dracocephalum komarovi,* which are widely used as folk medicines in Asia, Europe, and African countries [40,41]. This extensive presence of UNA in medicinal herbs suggests that UNA itself can exert therapeutic potential against diseases such as cancer and infectious protozoa.

## 5. Anticancer Effects

**Figure 2:**
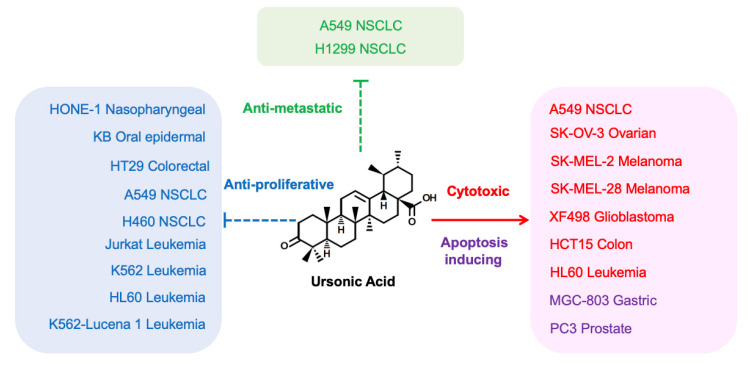
Anticancer effects of ursonic acid (UNA). UNA is known to exert various anticancer effects. It was reported that UNA inhibited proliferation of HONE-1 nasopharyngeal cancer cells, KB oral epidermal cancer cells, HT29 colorectal cancer cells, nonsmall cell lung cancer (NSCLC) (A549, H460) cells, and leukemia (Jurkat, K562, HL60, and K562-Lucena 1) cells. UNA showed cytotoxic effects in A549 NSCLC cells, SK-OV-3 ovarian cancer cells, SK-MEL-2 melanoma cells, SK-MEL-28 melanoma cells, XF498 glioblastoma cells, HCT15 colon cancer cells, and HL60 leukemia cells. UNA also induced apoptosis in MGC-803 gastric cancer cells and PC3 prostate cancer cells. Most recently, antimetastatic effects of UNA were reported in A549 and H1299 human NSCLC cells.

Cancer is a deadly disease, representing the second leading cause of death in the United States [42]. Major hallmarks of cancer include sustained proliferation and inhibition of apoptosis, which can be accomplished by modulation of molecular mechanisms in cancer cells [43]. Various cellular mechanisms of resistance to existing anticancer agents disrupt the efficacy of modern chemotherapy, indicating that development of new bioactive molecules is needed to solve this problem [44]. Triterpenoids are considered constituents of anticancer reagents [45]. Previous studies consistently reported that UNA demonstrates cytotoxicity to cancer cells originating from several tissue types (Figure 2). Ryu et al. reported cytotoxic effects of UNA against A549 nonsmall cell lung cancer (NSCLC) cells (IC_50_ = 7.7 μM), SK-OV-3 ovarian cancer cells (IC_50_ = 32.1 μM), SK-MEL-28 melanoma cells (IC_50_ = 22.2 μM), XF498 glioblastoma cells (IC_50_ = 25.9 μM), and HCT15 colon cancer cell lines (IC_50_ = 4.6 μM) [46]. In other studies, UNA effectively exerted antiproliferative effects in HONE-1 nasopharyngeal cancer cells, KB oral epidermoid cancer cells, and HT29 colorectal cancer cells with low IC_50_ values of 5.2 μM, 4.0 μM, and 6.3 μM, respectively [20]. UNA also impeded the proliferation of A549 (IC_50_ = 23.6 μM) and H460 (IC_50_ = 17.6 μM) lung cancer, and Jurkat (IC_50_ = 23.9 μM), K562 (IC_50_ = 12 μM), HL60 (IC_50_ = 12.8 μM), and K562-Lucena 1(IC_50_ = 12.6 μM) leukemia cells in vitro [47]. These results were comparable with the IC_50_ values of ULA in same cancer cells, which were 8.8 μM, 8.2 μM, and 4.7 μM, respectively [20]. Cytotoxicity of UNA was also shown in other cancer cells, including B16F0 murine melanoma (IC_50_ = 19.8 μM), L1210 murine leukemia (IC_50_ = 6.9 μM), SK-MEL-2 human melanoma (IC_50_ = 7.6 μM), and HL60 human leukemia cells (IC_50_ = 54.9 μM) [24,48,49]. UNA significantly increased survival days of mice bearing S180 sarcoma cells by seven days compared to nontreated mice, and this antitumor effect of UNA was reported to be correlated with the inhibition of topoisomerase-I (IC_50_ = 5.5 mM), one of targets for cancer treatment [48,50]. Furthermore, UNA isolated from *Toona sinensis* reduced the viability of several cancer cell lines and induced apoptosis of MGC-803 gastric cancer cells and PC3 prostate cancer cells [35]. Noticeably, UNA exerted a relatively weaker cytotoxic effect in NIH3T3 noncancer murine fibroblast cells (IC_50_ > 50 μM), whereas the IC_50_ of UNA on cancer cells ranged from 13.6 μM to 26.5 μM, suggesting that UNA can selectively reduce the survival of cancer cells while minimizing side effects against normal cells. UNA also repressed human cathepsin B for 49% at 20 μM [51]. Since cathepsin B plays a critical role in the proliferation and metastasis of tumors, it is expected that UNA may facilitate chemotherapy of cancer by targeting cathepsin B of cancer cells [52]. In studies regarding both UNA and ULA, cell viability and proliferation assays revealed that the antitumor effects of UNA were similar to, or stronger than, those of ULA [20,24,35,46,47,48,49,53,54]. Of note, in HCT15 colon cancer cells, the effective dose of UNA to exert cytotoxicity was 1.23 μM, which was remarkably lower than the concentration of ULA (IC_50_ > 20 μM) and the well-known anticancer agent Cisplatin (IC_50_ = 10.52 μM) [49]. These reports consistently demonstrated that UNA has strong antitumor potential comparable with ULA.

Metastasis occurs when cancer cells from primary tumors spread to other organs. Metastasis is often fatal to cancer patients, as it is responsible for approximately 66.7% of cancer deaths [55]. The extracellular matrix (ECM) holds cancer cells to the origin of oncogenesis as a biochemical and biophysical obstacle. For cancer cells to reach out to a great distance throughout the body, the ECM must be destroyed. The ECM is composed of various molecules, such as polysaccharides and proteins. Matrix metalloproteinases (MMPs) are a group of proteases that enzymatically digest ECM-composing proteins, such as gelatin and collagen. In addition, MMPs are known to promote cell migration nonproteolytically via their hemopexin domain. Therefore, metastasis of cancer is accelerated by migration and invasion, which can be promoted by MMPs in both enzymatic and nonenzymatic ways [56,57,58]. UNA was shown to significantly inhibit the invasion of A549 and H1299 human NSCLC cells (Figure 2) [59]. This antimetastatic effect of UNA is connected with suppressed proteinase activities of gelatinase MMP-2 and MMP-9 through transcriptional regulation. These inhibitory effects of UNA are similar to those of ULA, as ULA also hampered invasion and expression of MMPs in lung cancer cells [60]. Taken together, UNA can be inferred to have promising potential as an anticancer medicine.

## 6. Antiprotozoan Effects

**Figure 3:**
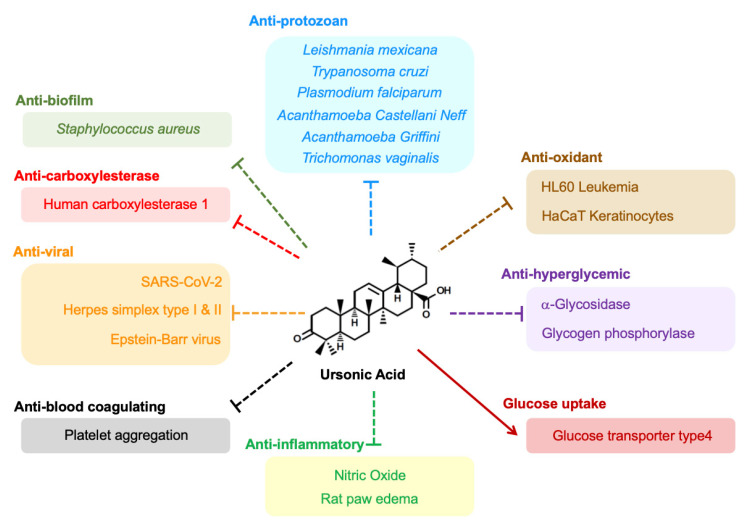
Antiprotozan and other therapeutic effects of ursonic acid (UNA). UNA has the potential to treat various human diseases. UNA shows antiprotozoan effects in *Leishmania mexicana*, *Trypanosoma cruzi*, *Acanthamoeba Castellanii Neff*, *Acanthamoeba Griffini*, *Trichomonas vaginalis,* and *Plasmodium falciparum*. UNA also has the possibility of improving hyperglycemia via inhibition of α-glycosidase and glycogen phosphorylase and stimulation of glucose transporter type 4. UNA also exhibits anti-inflammatory effects, such as reduction of nitric oxide, and inhibits rat paw edema. In addition, antiblood-coagulating effects of UNA are also reported. UNA is predicted to strongly bind with nsp15, indicating that UNA may inhibit the metabolism of SARS-CoV-2. UNA also exerts inhibitory effects in herpes simplex type I and II and Epstein–Barr viruses. Moreover, antioxidant activities of UNA are documented. Other bioactivities, including inhibition of human carboxylesterase 1 and biofilm formation of *Staphylococcus aureus,* are also reported.

UNA also has the potential to treat various human diseases other than cancer (Figure 3). Protozoa are composed of a wide range of unicellular living organisms, and there are diverse types of protozoa which can infect humans through various ways of transmission, such as direct, human-to-human, and fecal–oral routes [61]. Major human diseases caused by protozoa are malaria, African trypanosomiasis, Chagas’ disease, leishmaniasis, and other intestinal protozoal diseases, such as amoebic dysentery and toxoplasmosis [61]. Protozoan-related diseases account for substantial morbidity and mortality, affecting 500 million of people worldwide [62]. It is more worrying that these parasites can develop resistance to existing antiprotozoan drugs through mutations of several proteins [61]. Hence, discovery of novel molecules is required to overcome this problem. It is known that plants possess a wide range of phytochemicals with antiprotozoan activities, indicating that there is the possibility of development of novel antiprotozoan agents [63]. Among them, ULA is reported to possess inhibitory activities against infection by protozoa [64]. Several studies highlighted that UNA showed inhibitory effects in parasitic protozoa as well. UNA has the potential to display inhibitory effects against *Leishmania* species, a protozoan that causes the tropical disease leishmaniasis [65]. UNA selectively repressed the proteinase activity of cathepsin L of *Leishmania mexicana* through a partially noncompetitive inhibition [51]. UNA demonstrated IC_50_ values of 3.78 µM, superior to the effect exerted by ULA (IC_50_ = 8.1 µM) [51,66]. It was also reported that UNA exerted trypanocidal activity against epimastigotes of *Trypanosoma cruzi*, a parasitic protozoan known to cause Chagas’ disease [41]. A minimum lethal concentration of UNA in *Trypanosoma cruzi* was 50 µM, while the concentration of ULA was 100 µM. UNA also exhibited amoebicidal activities against *Acanthamoeba Castellanii Neff* (IC_50_ = 34.9 µM) and *Acanthamoeba Griffini* (IC_50_ = 11.1 µM), which can cause *Acanthamoeba* keratitis and granulomatous amebic encephalitis [67]. This study was notable in that there was low cytotoxicity of UNA in J774.A1 murine macrophages (IC_50_ > 100 µM) compared to ULA (IC_50_ = 17.7 µM) and the antiseptic agent chlorhexidine (IC_50_ = 7.4 µM), indicating the possibility of UNA to selectively suppresses *Acanthamoeba* spp. without affecting mammalian cells [67]. Moreover, UNA was shown to exert an in vitro suppressive effect in *Trichomonas vaginalis,* which is responsible for trichomoniasis, a sexually transmitted disease (55% inhibition at a dose of 100 µM) [68]. In addition, UNA showed an antimalarial effect against a chloroquine-resistant strain of *Plasmodium falciparum* in a micromolar dosage (IC_50_ = 41 µM), which was slightly more active than the effect from ULA (IC_50_ = 53 µM) [69]. It is noteworthy that UNA exhibited suppressive effects in different types of parasitic protozoa, exhibiting IC_50_ values with micromolar concentrations.

## 7. Antihyperglycemic Effects

Hyperglycemia, which is highly associated with diabetes mellitus and acute stress, is a condition in which cellular glucose uptake fails and blood glucose levels aberrantly increase [70,71]. Increased blood glucose levels can elevate mortality, as this can impair immune responses, promote inflammation, and worsen cancer progression [71,72]. Pentacyclic triterpenoids have the potential to control hyperglycemia, as they upregulate glucose uptake in skeletal muscle cells [73,74]. In a previous study, UNA inhibited the activity of yeast α-glucosidase in vitro (IC_50_ = 101 µM) more effectively than the FDA-approved antidiabetic drug acarbose (IC_50_ = 236.3 µM) [40]. UNA inhibited biological activity of rabbit muscle glycogen phosphorylase a, which is closely linked with blood glucse control (IC_50_ = 57 µM) [75,76]. In another study, UNA effectively inhibited α-glucosidase of baker’s yeast (IC_50_ = 2.47 µM), and this suppression was significantly stronger than that by ULA (IC_50_ = 5.08 µM) and acarbose (IC_50_ = 573.5 µM) [77]. It was also reported that UNA increased protein expression of glucose transporter type 4 (GLUT4) in the plasma membrane and promoted glucose uptake in L6 rat skeletal muscle cells, without affecting cell viability, while ULA showed no significant effect and reduced cell viability [25]. ULA is also well-known for its antihyperglycemic effects [19]. In some contexts, ULA could inhibit α-glucosidase (IC_50_ = 8.4 µM) more strongly than UNA (IC_50_ = 101 µM) in vitro [40]. Also, dietary supplementation (0.01 and 0.05 g per 100 g of diet) and oral administration (200 and 300 mg/kg per day) of ULA successfully lowered blood glucose levels of diabetic mice and rats, respectively [40,78]. However, there is a possibility that ULA cannot upregulate the transportation of glucose in some contexts [25]. In preparation of this case, UNA could be developed as a complimentary drug for protection from, and improvement of, hyperglycemia.

## 8. Anti-Inflammatory Effects

While inflammation is responsible for numerous diseases, including arthritis, diabetes, multiple sclerosis, Alzheimer’s disease, and Parkinson’s disease, it is also highly associated with cancer progression [6,79]. Anti-inflammatory effects of triterpenoids from medicinal plants are well reported [6]. Nitric oxide (NO) is a molecule which is highly linked with immunity and inflammation [80]. In a recent study, UNA remarkably inhibited production of NO in lipopolysaccharide (LPS)-induced RAW264.7 murine macrophage cells in the low micromolar range (IC_50_ = 4.94 µM), whereas ULA showed no repressive action [22]. Notably, the keto group of UNA at the C-3 position led to significantly different anti-inflammatory activity. Also, UNA effectively inhibited human platelet aggregation induced by arachidonic acid (IC_50_ = 0.26 mM) and exerted an anti-inflammatory effect in rat paw edema induced by carrageenan (53% of inhibition at dose of 50 mg/kg) [36]. These inhibitory effects of UNA were comparable with quercetin (IC_50_ = 4.41 µM) and aspirin (28–71% of inhibition at 150–300 mg/kg), which are well-known anti-inflammatory molecules [22,36]. Although ULA is well-known for its anti-inflammatory effects, it can also exert proinflammatory activities [9,22], indicating that UNA may be used as an alternative drug for the amelioration of inflammatory diseases.

## 9. Antiviral Effects

It was reported that several types of triterpenoids can exhibit antiviral activities [81,82,83]. Earlier studies highlighted that UNA possesses antiviral potential as well. In previous research, UNA suppressed cytopathic effects of herpes simplex virus types I and II infecting Vero monkey kidney cells [34,84]. In particular, the inhibitory effect of UNA (IC_50_ = 17.6 µM) against herpes simplex virus type I was stronger than the antiviral effect of ULA (IC_50_ = 175.1 µM) [84]. Another study reported that UNA significantly enhanced the inhibition of Epstein–Barr virus (EBV) early antigen induction and cytotoxicity of ULA in Raji–Burkitt’s lymphoma cells [85]. While ULA inhibited about 20% of EBV induction at a dose of 6.5 µM and did not reduce cell viability, 6.5 µM of UNA completely suppressed EBV induction, and cell survival decreased to 20% at 65 µM [85]. This research also demonstrated the keto group at the C-3 position of UNA for increased biological activities [85]. There was an attempt to design a theoretical model of a carbon nanotube–UNA drug matrix to block the entry of human immunodeficiency virus (HIV) [86]. Moreover, a recent document demonstrated that UNA has the potential to strongly interact with severe acute respiratory syndrome coronavirus 2 (SARS-CoV-2) Nsp15, an endoribonuclease which is essential for the lifecycle of coronaviruses [87,88]. Molecular docking and dynamics analyses show that UNA is predicted to bind stably with the catalytic residues of SARS-CoV-2 Nsp15 via hydrogen bonds, suggesting that UNA may represent one of drug candidates to inhibit replication of SARS-CoV-2, the virus responsible for the coronavirus disease 2019 (COVID-19) pandemic.

## 10. Antioxidant Effects

Oxidative stress, which is induced by reactive oxygen species (ROS), can affect various cellular events such as cancer proliferation, inflammation, and aging [89]. Some studies conducted assays regarding the antioxidant activity of UNA. In a previous report, UNA effectively reduced ROS levels of HL60 leukemia cells (IC_50_ = 55 µM), although this effect was weaker than the effect of ULA (IC_50_ = 26.2 µM) [24]. Another study showed that UNA could diminish intracellular ROS levels of HaCaT keratinocytes (40% inhibition at 10 µM), which is correlated with downregulated transcription of interstitial collagenase MMP-1 [59]. However, in the same report, UNA did not change the redox levels of A549 and H1299 NSCLC cells [59]. These conflicting results imply that UNA’s antioxidant effects may be dependent on the type of cell. Additional investigations are needed to check the antioxidant potential of UNA in cells from different tissue origins.

## 11. Other Expected Therapeutic Potential

Human carboxylesterase 1 (hCE1), a serine hydrolase distributed in liver and adipocytes, is highly associated with type 2 diabetes and hypertriglyceridemia [90]. It was reported that UNA displayed strong inhibitory activity against hCE1 (IC_50_ = 0.037 µM), and this effect was more powerful than the activity of ULA (IC_50_ = 0.24 µM) [90].

Biofilm is one of major factors that lowers the efficacy of antibacterial drugs. In a previous study, UNA showed antibiofilm activity of *Staphylococcus aureus* (82% inhibition at 100 µM), although it did not exert antibacterial activity compared to ULA [91]. UNA has relatively lower cytotoxicity against Vero monkey kidney cells (IC_50_ > 100 µM), whereas ULA significantly reduced cell viability (IC_50_ < 5 µM), indicating that UNA may be developed as a safer antibiofilm reagent [91].

## 12. Modulation of Cell Signaling Pathways by Ursonic Acid

**Figure 4:**
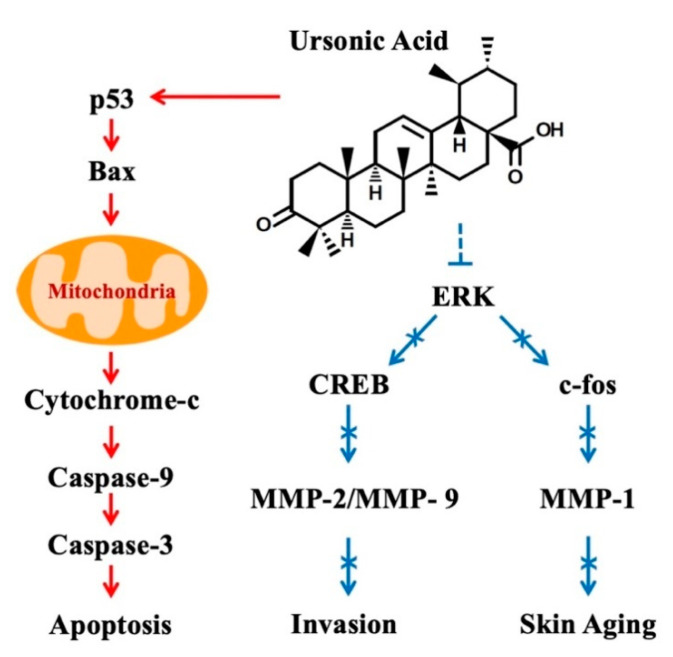
Summary of signaling pathways affected by ursonic acid (UNA). UNA induces apoptosis through the mediation of the mitochondria pathway in MGC-803 gastric cancer cells. On the other hand, UNA exerts antimetastatic effects via inhibition of ERK–CREB signaling. This regulation results in the downregulation of matrix metalloproteinase (MMP)-2 and MMP-9, and attenuation of invasion of A549 and H1299 NSCLC cells. UNA also negatively regulates the ERK-c-fos signaling pathway, leading to the inhibition of MMP-1 and possible suppression of skin aging.

Most cellular activities involve complex signaling pathways, including apoptotic pathways and mitogen-activated protein kinase (MAPK) pathways. In intrinsic apoptotic signaling pathways, tumor suppressors such as p53 induce apoptosis via regulation of proapoptotic proteins (BIM, PUMA, NOXA, and BAX) and antiapoptotic proteins (BCL-2, MCL-1, and BCL-XL) [92]. MAPKs are composed of a cascade of several kinases to transmit cellular signals to diverse types of transcription factors [93]. As MAPK signaling pathways can activate or inactivate cell metabolisms, such as cell proliferation and apoptosis, MAPKs are one of major targets for cancer treatment. The demonstrated anticancer effects of UNA, which were consistent with various scientific investigations, imply that UNA may be able to regulate cancer-related signaling pathways, including apoptotic and MAPK pathways. Unfortunately, detailed mechanisms of UNA’s pharmacological potential have not yet been sufficiently explained. Two publications previously explained the signaling pathways of cancer cells regulated by UNA (Figure 4). In MGC-803 human gastric cancer cells, UNA increased the protein levels of p53, cell death effector BAX, and activated caspase-3 and caspase-9, which execute apoptosis of cells [35]. In A549 and H1299 human lung cancer cells, UNA inactivated phosphorylation extracellular signal-regulated kinase (ERK) and transcription factor cAMP response element-binding protein (CREB), which are highly associated with the expression of MMPs and metastases [59,94,95,96]. In HaCaT keratinocytes, UNA inhibited ERK and the c-fos signaling pathway and repressed the expression of collagenase MMP-1, which is a principal enzyme responsible for skin aging [59,97]. Several research studies further showed that UNA exerted strong pharmaceutical effects in diverse types of cells; further research may unveil additional antitumour, as well as other, therapeutic mechanisms exerted by UNA. Particularly, it is expected that UNA may strongly affect signaling pathways in some contexts, such as cytotoxicity in HCT15 colon cancer cells (IC_50_ = 1.23 μM) and attenuated NO production in RAW264.7 macrophages (IC_50_ = 4.94 µM) [22,49]. Furthermore, previous in silico assays predicted that UNA could display inhibitory activity against the transcription factor NF-κB, with a probability of activation value of 54% [98]. This implies that UNA may be involved in regulation of NF-κB signaling pathways, which are responsible for proinflammatory responses [98].

## 13. Modifications of Ursonic Acid

Despite the strong pharmacological potential of UNA, there is a disadvantage that triterpenoids, including UNA, have poor water solubility, which is one of important parameters for bioavailability parameters, such as absorption, permeation, first effects, and elimination of drugs [99]. As UNA is an oxidized derivative of ULA, there is a high probability that UNA may also meet this obstacle. This factor should be considered, since poor bioavailability can result in an abnormal gap between in vitro, in vivo, and clinical studies. Therefore, molecular modifications of UNA may be needed to improve this limitation. In a recent study, the C-2 position of UNA was brominated by an organic reaction using copper (II) bromide [100]. This brominated derivative of UNA (2α/2β-bromo UNA) showed cytotoxicity in various types of cancer cells, such as CCRP–CEM leukemia cells (IC_50_ = 3.6 µM), and induced cellular apoptosis, mitochondria depolarization, and cell cycle arrest. Also, 2α/2β-bromo UNA showed high solubility in water and stability in human plasma, indicating high bioavailability for in vivo tests [100].

Modifications of UNA could be conducted to improve its pharmacological potential. In another work, derivatives of UNA generated by reaction with oxalyl chloride and various phenyl amines inhibited tumor necrosis factor-α (TNF-α)-induced nuclear factor-kappa B (NF-κB) activation of H460 human lung cancer cells, with values of IC_50_ ranging from 0.94 to 15 µM [101]. Some UNA derivatives with substitution at the C-2 position suppressed the catalytic activity of baker’s yeast α-glucosidase (IC_50_ from 0.71 to 10.32 µM), while the IC_50_ values of ULA and UNA were 5.08 and 2.47 µM, respectively [102].

Oher derivatives of UNA, which are synthesized by reaction with organic compounds such as potassium thiocyanate, alkyl ammonium acetate, and o-aminobenzaldehyde, are well documented [100,103]. However, these analogues, which underwent removal of the keto group at the C-3 position with further modifications, displayed relatively weaker anticancer effects [100]. It is assumed that a keto group at the C-3 position is a crucial factor for preserving the pharmaceutical potential of UNA.

## 14. Future Perspectives

Results from previous publications demonstrated that UNA could be a strong drug candidate to treat human diseases. However, there is a limitation in that most of experiments regarding UNA were preliminary and lacked profound assays, while the pharmaceutical effects of ULA were observed from various in vivo tests and its complex mechanisms of action were well explained [19]. Since there are diverse types of cancer cells suppressed by UNA, in vivo experiments using tumor xenograft animal models would be helpful to confirm these anticancer effects. Furthermore, analyses of cellular events, such as metastatic abilities, apoptosis, protein expression, and transcriptional expression, may be studied to elucidate cellular and molecular mechanisms of UNA. As UNA could attenuate enzymatic activities of some proteins, such as α-glucosidase, cathepsin L of *Leishmania Mexicana,* and hCE1, studies of the interactions between UNA and various proteins may facilitate understanding of the pharmacodynamics of UNA as a drug candidate. Other attempts, including molecular modifications and cotreatment with other well-known compounds, may be conducted to enhance the bioactivities of UNA.

## 15. Conclusions

In conclusion, UNA, a phytochemical from medicinal herbs, has therapeutic potentials that are similar to, or stronger than, ULA in some aspects. In particular, UNA showed strong potential anticancer, antiproliferative, and antiprotozoan effects. However, in contrast to ULA, scientific studies regarding UNA’s therapeutic activities are still lacking. Since most assays concerning UNA were conducted in vitro, additional in vivo experiments may be required to confirm these potential pharmaceutical effects. Moreover, detailed studies to elucidate the molecular and cellular mechanisms are needed to help understanding of activities exerted by UNA. Further analyses and applications may facilitate the possibility of UNA being developed in new medications to treat various human diseases.

## Abbreviations

UNA	Ursonic acid
ULA	Ursolic acid
NF-κB	Nuclear factor-κB
IPP	Isopentenyl diphosphate
DMAPP	Dimethylallyl diphosphate
NSCLC	Nonsmall cell lung cancer
MMP	Matrix metalloproteinase
NO	Nitric oxide
LPS	Lipopolysaccharide
EBV	Epstein–Barr virus
HIV	Human immunodeficiency virus
SARS-CoV-2	Severe acute respiratory syndrome coronavirus 2
COVID-19	Coronavirus disease 2019
hCE1	Human carboxylesterase 1
MAPK	Mitogen-activated protein kinase
ERK	Extracellular signal-regulated kinase
CREB	cAMP response element-binding protein
ROS	Reactive oxygen species
TNF-α	Tumor necrosis factor-α
